# Postoperative Infections in Hepatopancreatobiliary Malignancy: A Tertiary Care Center Experience

**DOI:** 10.7759/cureus.43449

**Published:** 2023-08-14

**Authors:** Basant Mahaddevappa, Rohith Muddasetty, Vinu Sankar V S

**Affiliations:** 1 Hepatopancreatobiliary Surgery and Liver Transplantation, Health Care Global (HCG) Hospital, Bangalore, IND

**Keywords:** percutaneous transhepatic biliary drainage (ptbd), pancreatic tumors, ercp stenting, gall bladder malignancy, obstructive jaundice, hilar tumour

## Abstract

Introduction: Wound infection following surgery is not an uncommon entity in any malignancy. Various factors play a role in the development of infection like diabetes, the duration of surgery, intraoperative blood loss, and prior stenting. Obstructive jaundice is a common presentation in hepatopancreatobiliary malignancy, and most of the patients are being stented to relieve jaundice. The role of preoperative stenting and biopsy in these malignancies is a debatable topic. These procedures have a negative impact on the postoperative outcome.

Materials and methods: We have retrospectively analyzed the patients who have undergone surgery involving biliary enteric anastomosis from January 2013 to June 2023, and the following results have been formulated after using appropriate statistical tests for the level of significance.

Results: One hundred and fifty patients had surgeries performed involving biliary enteric anastomosis, with M:F=14:11 and a mean age of 57.8 years (standard deviation (SD): 9.6). On performing binary regression analysis using various parameters. Preoperative stenting increases the risk of the growth of bacteria in bile by 12 times (OR: 12, 95% CI: 5.25-27.42, p<0.001) and the presence of bacteria in bile increased the risk of wound infection by 16.5 times (OR: 45, 95% CI: 7-38.89, p<0.001). The duration of hospital stay was significantly longer in patients who developed wound infections, thus increasing the cost of treatment and delaying the initiation of adjuvant treatment.

Conclusion: Various factors play a role in the development of wound infections following any surgery. From the analysis performed, we found that the duration of surgery and preoperative procedures in the form of stenting increased the risk of growing bacteria in the bile, which later increased the risk of developing a wound infection. Wound infections prolong the hospital stay and delay the initiation of adjuvant treatment. Thus, preoperative stenting should be performed after discussion in a multidisciplinary tumor board meeting.

## Introduction

Obstructive jaundice is a common presentation in hepatopancreatobiliary malignancies. Relief from jaundice has several advantages like improving liver function, coagulopathy, and hemorrhagic diathesis [1]. Biliary stenting does not reduce circulating endotoxins and cytokines, while all stented patients had positive bile cultures [2]. Biliary stenting is not without drawbacks; a few of these include an increased risk of wound complications, infection entering a previously sterile system, and an increased length of hospital stay [3]. The debate about preoperative biliary stenting in resectable malignancy is now limited to a few scenarios like high bilirubin values where the cut-off bilirubin value has not been established. There are absolute indications for which preoperative biliary stenting has been advocated in cases of distal biliary obstruction-metastatic disease for palliative chemotherapy, borderline resectable malignancy planning for neoadjuvant chemotherapy, severe cholangitis not responding to antibiotics, patients with cardiopulmonary disease or acute kidney injury that requires stabilization. In proximal biliary obstruction, biliary stenting is required when major hepatectomy is anticipated. More and more patients are using preoperative biliary stenting, with over 77% of them getting stented before seeing a surgeon [4]. We investigated the patients who underwent hepatopancreatobiliary surgery at our tertiary care center facility, looking at the association between various parameters, which included age, diabetic status, duration of surgery, intraoperative blood loss, and preoperative biliary stenting and biopsy with development of wound infection [5].

## Materials and methods

We have performed a retrospective analysis of our patients who were admitted for hepatopancreatobiliary malignancies and underwent surgery involving biliary enteric anastomosis. We documented the history and demographic details of the patients, the indication for surgery, the surgery performed, bile culture growth and sensitivity patterns, the duration of surgery, intraoperative blood loss, and the development of wound infection.

As a routine, we would aspirate the bile from the gall bladder using an 18G needle (Romsons, Noida, India) as soon as laparotomy is performed for bacterial culture and sensitivity patterns. The surgeries performed were divided into three groups for analysis: Whipple pancreatoduodenectomy, hepatectomy-common bile duct (CBD) excision-biliary enteric anastomosis, and palliative bypass. Radical cholecystectomy with biliary tree excision was included in the category of hepatectomy-CBD excision-biliary enteric anastomosis. Before beginning a definitive surgery, we perform a diagnostic laparoscopy to rule out disseminated disease. Wound infection is defined by ASEPSIS criteria, which include erythema, serous or purulent discharge, separation of deep tissues, isolation of bacteria, need for additional treatment, and prolongation of hospital stay for more than 14 days [6]. All data were documented in a Microsoft Excel sheet (Microsoft Corp., New York, USA); continuous variables were represented using the mean and standard deviation (SD), while categorical variables were represented as numbers and percentages. Various parameters were evaluated for their relation to the outcome (wound infection), and levels of significance were measured using univariate and multivariate binary logistic regression analysis, the Chi-square test for categorical data, and the t-test for means. A P-value less than 0.05 was considered significant.

## Results

The study was conducted during the period of January 2013 to June 2023. A total of 150 patients underwent surgery for hepatopancreatobiliary malignancies; among them, 84 were males and 66 females (M:F=14:11). The mean age of the patients was 57.8 years (SD: 9.6). Indications for surgery were adenocarcinoma of the ampulla, head of the pancreas, and distal CBD in 108 patients; neuroendocrine tumor of the pancreatic head in 15 patients; carcinoma gall bladder with common bile duct (CBD) involvement in 12 patients; perihilar cholangiocarcinoma in nine patients; and cystic neoplasm of the head of the pancreas in six patients. One hundred seventeen patients underwent Whipple pancreatoduodenectomy, 18 patients underwent hepatectomy-CBD excision-biliary enteric anastomosis, and 15 patients underwent palliative bypass. Sixty out of 150 patients had a preoperative procedure in the form of a biopsy (33 patients) or biliary stenting (45 patients). Biliary stenting was in the form of endoscopic retrograde cholangiopancreatography (ERCP) with a plastic stent in 27 patients, ERCP with metallic stenting in 12 patients, and percutaneous transhepatic biliary drainage (PTBD) using a plastic catheter in six patients. Demographic details are summarized in Table 1.

**Table 1:** Demographic data of the patients. CBD: common bile duct, ERCP: endoscopic retrograde cholangiopancreatography, PTBD: percutaneous transhepatic biliary drainage, SD: standard deviation.

Parameters	Values
Total number of patients	150
Sex	
Male	84
Female	66
Age in years	
Mean (SD)	57.8 (9.6)
Diabetics	63
Duration of surgery in hours	
Mean (SD)	5.62 (1.87)
Intraoperative blood loss in mL	
Mean (SD)	233 (223.5)
Procedures performed	
Whipple pancreatoduodenectomy	117
Hepatectomy-CBD excision-biliary enteric anastomosis	18
Palliative bypass	15
Number of patients who undergone preoperative procedures	60
Biopsy alone	33
Biliary stenting alone	45
Duration of hospital stay in days	
Mean (SD)	12.68 (6.3)

Risks of wound infection among the patients were evaluated across various parameters, which are summarized in Table 2. The binary logistic regression shows that the association of preoperative biopsy with wound infection is statistically significant (p=0.004), i.e., the patients for whom preoperative biopsy has been done have 0.16 times less chance for wound infection than compared to the patients for whom preoperative biopsy is not done. Even on multivariate analysis, it was found that preoperative biopsy was associated with 0.019 times less risk of wound infection. The association between preoperative stenting and wound infection is statistically significant (p<0.001), i.e., the patients who have undergone preoperative stenting had 3.85 times more chance for wound infection than compared to the patients who had not undergone preoperative stenting. However, on multivariate analysis, there was no significant association identified between stenting and wound infection. The association between growth of organisms in bile and wound infection is statistically significant (p<0.001) in both univariate and multivariate analysis, i.e., the patients with growth of organisms in bile had 16.50 times more chance for wound infection than compared to the patients who did not have growth of organisms in bile. The association between the duration of surgery and wound infection is statistically significant (p=0.029) on univariate analysis but did not reach statistical significance on multivariate analysis. The duration of hospital stay was also significantly longer in the patients with wound infections (15.1±6.2 days vs 11.5±6 days; p=0.029).

**Table 2:** Risk of infection with respect to various parameters. OR: odds ratio, CI: confidence interval.

Predictor variable	Number of patients	Univariate analysis			Multivariate analysis		
		OR	95% CI for OR	p-value	OR	95% CI for OR	p-value
Age	150	0.976	(0.940, 1.01)	0.186	0.966	(0.910, 1.02)	0.250
Diabetes mellitus	63	0.760	(0.376, 1.53)	0.44	0.408	(0.120, 1.38)	0.151
Duration of surgery	150	0.786	(0.634, 0.975)	0.029	0.722	(0.502, 1.038)	0.078
Blood loss	150	1	(0.99, 1.002)	0.451	1	(0.997, 1.003)	0.938
Preoperative procedure	60	1.83	(0.914, 3.67)	0.08	-	-	0.999
Preoperative biopsy	33	0.160	(0.046, 0.55)	0.004	0.019	(0.002, 0.169)	<0.001
Preoperative stenting	45	3.85	(1.83, 8.10)	<0.001	-	-	0.998
Growth of organisms in bile	45	16.50	(7, 38.89)	<0.001	43.64	(9.13, 208.53)	<0.001

The risks of growing organisms in the bile obtained during surgery were evaluated, and results are tabulated in Table 3. The association between preoperative procedure and bacterial growth in bile is statistically significant (p<0.001), i.e., the patients who have undergone preoperative procedure have 7.94 times more chance for bacterial growth in bile than the patients who have not undergone preoperative procedure. The association between preoperative stenting and bacterial growth in bile is statistically significant (p<0.001), i.e., patients who have undergone preoperative stenting have a 12 times higher chance of bacterial growth in bile than patients who had no preoperative stenting performed. However, on multivariate analysis, none of these parameters were significantly associated with the growth of bacteria in the bile.

**Table 3:** Risk factors for the growth of organisms in the bile. OR: odds ratio, CI: confidence interval.

Predictor variables	Number of patients	Univariate analysis			Multivariate analysis		
		OR	95% CI for OR	p-value	OR	95% CI for OR	p-value
Diabetes mellitus	63	1.93	(0.954, 3.92)	0.067	1.24	(0.527, 2.95)	0.614
Preoperative procedure	60	7.94	(3.59, 17.54)	<0.001	5.76	(0.851, 39.05)	0.073
Preoperative biopsy	33	1.45	(0.643, 3.28)	0.368	0.278	(0.076, 1.02)	0.054
Preoperative stenting	45	12	(5.25, 27.42)	<0.001	3.78	(0.778, 18.39)	0.099

The organisms grown were as follows: *Escherichia coli* in 27 patients, *Klebsiella pneumonia* in 15 patients, Pseudomonas in three patients, and other Enterococci species in three patients. The relationship between organisms grown and wound infection has been summarized in Table 4. There was no significant risk of wound infection with different types of organisms grown. These organisms were often sensitive to one of the following drugs: meropenem, amikacin, tigecycline, and cefepime.

**Table 4:** Summary of organism growth in bile and wound infection.

Organism grown	Number of patients	Wound infection	p-value
E. coli	27	21	0.34
K. pneumonia	15	9	
Pseudomonas	3	2	
Enterococci species	3	1	

## Discussion

Preoperative biopsy is not necessary for resectable hepatopancreatobiliary malignancies, according to National Comprehensive Cancer Network (NCCN) guidelines. The biopsy is required when neoadjuvant or palliative treatment is planned [7,8]. Not all patients with hepatopancreatobiliary cancers require preoperative biliary stenting. There are several articles supporting and opposing biliary stenting. Jagannath et al. reported that positive intraoperative bile cultures were associated with higher morbidity and mortality following pancreatoduodenectomy, and a higher number of patients had positive bile cultures after stenting [9]. van der Gaag et al. evaluated patients undergoing pancreatic head resections following preoperative biliary stenting in the form of ERCP or PTBD and found an increased rate of complications [10]. Gavazzi et al., in their article, suggested the selective use of preoperative biliary stenting in view of the increased risk of surgical site infections [3]. Preoperative biliary stenting increases the incidence of general complications and wound infections, according to Scheufele et al.'s meta-analysis of 25 studies [11]. In perihilar malignancies, preoperative biliary drainage is advocated when major hepatectomy has been planned in conjugation with portal vein embolization [12]. Endoscopic nasobiliary drainage has been performed in various centers with a lower risk of biliary contamination; however, none of our patients had undergone this procedure.

In our study, we have seen that patients developed a growth in the bile culture, which was significantly higher among the patients who underwent a biopsy or biliary stenting procedure. Being a tertiary center, we get most of our patients referred from other primary and secondary care centers. The patients who were stented at the time of referral lacked a clear indication. We have seen a higher chance of growth in bile cultures and the development of wound infections in those patients who were stented. The growth in bile also correlated with an increased occurrence of wound infections. These organisms have shown good sensitivity to meropenem, amikacin, and cefepime at our institution. Thus, we have initiated an antibiotic protocol of adding preoperative intravenous meropenem 1 g in those patients who are stented based on the pattern of sensitivity we have obtained in our study.

The duration of surgery increases the risk of wound infection by increasing exposure to potential contamination and lowering the tissue antibiotic concentration [13]. We have not found any significance with respect to wound infection and duration of surgery. We would like to stress the fact that patients being operated on for >8 hours in our study had no wound infection because most of them underwent a laparoscopic or robotic procedure and tend to have small incisions and minimal tissue trauma. The duration of hospital stay increases concurrently as the patient develops a wound infection, thus increasing the cost of treatment and delaying the initiation of adjuvant therapy.

Preoperative biliary stenting or biopsy for a patient presenting with obstructive jaundice due to hepatopancreatobiliary malignancy is to be considered only after a thorough evaluation of the resectability of the lesion and after discussion in a multidisciplinary tumor board. Stenting increases the risk of bacterial growth in the bile and wound infections, thus increasing hospital stays, the need for escalation of antibiotics, and the delay in treatment. The limitations of our study are that it is retrospective in nature; stenting was not performed in our institution on most of the patients.

## Conclusions

Preoperative procedure in the form of a biopsy or stenting to relieve jaundice has been shown to be associated with an increased risk of wound infections. Although the topic of preoperative biliary stenting is less controversial nowadays, we still come across patients undergoing biliary stenting without a review in a multidisciplinary tumor board. A biliary stenting procedure can increase the risk of bacterial growth in the bile, wound infections, the need for prolonged antibiotics, a longer hospital stay, increased costs, and a delay in the initiation of adjuvant treatment. Thus, we conclude that preoperative biliary stenting should be used selectively.
